# Supplementary material for: BOLD activity during emotion reappraisal positively correlates with dietary self-control success
Source: Soc Cogn Affect Neurosci. 2020 Jul 23;18(1):nsaa097. doi: 10.1093/scan/nsaa097 (PMC9910278; doi:10.1093/scan/nsaa097)
Supplement: File010_nsaa097 [file file010_nsaa097.docx]

**Supplementary Online Material

BOLD activity during emotion reappraisal positively correlates with dietary self-control success**

Silvia U. Maier^1,2,3^, Todd A. Hare^1,2^

^1^ Zurich Center for Neuroeconomics, Department of Economics, University of Zurich

^2^ Neuroscience Center Zurich, University of Zurich, Swiss Federal Institute of Technology Zurich

^3^ Translational Neuromodeling Unit, Institute for Biomedical Engineering, University of Zurich and ETH Zurich

**Supplementary Methods**

***Emotion regulation success across valence domains***

To test whether emotion regulation was equally successful across stimuli with positive and negative valence, we estimated a regression model according to equation S1 below

Eq. (S1) regulation success = β_0_+ β_1_ type + ε

In this model, *regulation success* was defined for negative-valence stimuli as the difference, Reappraisal minus View, because the reappraised rating should be higher (i.e. more positive) than the unregulated viewing rating if reappraisal of negative stimuli was successful. The difference, View minus Reappraise, described success in the positive reappraisal trials, because for positive stimuli the unregulated View ratings should be higher than the reappraised rating when successfully modulating positive emotions. Trial *type* was a factor with 2 levels (1 = negative, 2 = positive valence). The model included subject-specific random intercepts and slopes for the trial type.

***Drift diffusion modeling***

In order to test whether there was a decision bias towards refusing foods, we modeled the data using a drift diffusion model that allows for different attribute onset times for taste and health. For full details on this modeling approach, we refer the reader to the paper by Maier et al. (in press, preprint available at bioRxiv (Maier et al., 2020)). Briefly, this model is a time-varying drift diffusion model (DDM) that is augmented by one parameter that captures the start time of health attribute processing relative to the processing start time of taste (parameter *RST*). This feature of the model allows for better identification of the weight quantifying the influence of each attribute on the evidence accumulation process and any starting-point bias. In our formulation of the model, a negative sign on the bias signifies that participants had a starting point bias in favor of refusing to eat the food.

We estimated this model on the food choices in the current dataset (N=39 participants who are included in the dietary self-control analyses, hereafter abbreviated as study *ESC*) and used two-sample BEST tests (Kruschke, 2013) to compare the starting-point bias (hereafter in short: *Bias*) and relative-health-start-time (*RST*) estimates to the results of (Hare et al., 2011) (hereafter abbreviated as study *IAC*). This study was employing the same choice setup of choosing the food on the screen versus nothing and also had a condition with a health reminder.

***Reaction time simulations***

In order to test whether reaction times generated by the DDM could yield the pattern of results observed in Table 4, we simulated reaction times for all trials using the best generating DDM parameters and the taste and health value difference from zero (i.e., when refusing to eat) for each participant. Corresponding to the choice boundary definition that was used in the fitting of the DDM, we recorded the corresponding simulated choices as “yes” if they had a positive sign, and as “no” if they had a negative sign. We took the absolute value of the simulated RTs and applied the natural logarithm in order to fit them using the regression model described in Eq. 4.

***Correlations between individuals’ BOLD responses and their DDM Bias parameters***

In order to test for correlations between BOLD responses and the Bias parameter, we ran a regression using all 6 DDM parameters and an intercept, because the DDM parameters are interdependent. Within this group-level regression model, we specified two contrasts testing for either a positive or a negative correlation with the Bias parameter. We tested whether the individual levels of these parameters explained individual variance in the BOLD signal of 1) the contrast “Self-Control Success > Self-Control Failure” in GLM-SCS and 2) of the contrast “All Choice” in GLM-FC.

**Supplementary Results**

***Drift diffusion model parameter comparison to previous results.***

We first tested whether the participants of the current study (ESC) expressed a bias towards refusing the foods. The results in Table S2 show a negative sign for the bias term, indicating that there was indeed a bias towards refusing the foods in the current study. The comparison with the study of Hare et al. (2011) showed that compared to the health-cue condition in IAC, participants in the current study expressed a greater bias towards saying “No” (difference in starting point bias = -0.156, Posterior Probability (PP) of (ESC bias < IAC health condition bias) = 0.98, 95% HDI = [-0.30; -0.01]). Note that, in our model formulation, negative bias parameters favor the “No” response. As expected, the difference in the bias terms across studies was even more pronounced when comparing to the natural choices in IAC (difference in starting point bias = -0.377, PP(ESC bias < IAC natural condition bias) = 0.999, 95% HDI = [-0.54; -0.22]). This indicates that across all trials in the present study participants showed a greater inclination towards refusing the foods, and that this bias was greater than could have been expected from the comparable study.

We also tested whether there were any differences in the attribute consideration onset timing (RST). Here, we did not observe differences between the ESC sample and the IAC health condition, which is most comparable to our present study setup where we also introduced a health reminder. The relative start time for health did not differ significantly between the ESC study and the IAC health condition: the mean difference in the RST parameter was -0.04 (PP(RST ESC > RST IAC health condition) = 0.39, 95% HDI = [-0.28; 0.21]).

***Simulation results from the DDM***

In order to test whether an overall bias toward responding “Not Eat” – which is present in the DDM parameters – can still lead to the reaction time pattern we observe in Figure 3, we simulated reaction times based on the best-fitting DDM parameters for each subject. The simulated RTs yielded a very similar pattern. Reaction times were faster when the simulated agents refused to eat the foods, except for the palatable-healthy foods where accept response was favored by the healthiness and palatability attributes (Supplementary Figure S2). These simulation results indicate that our empirical results are consistent with an overall bias toward refusing to eat the foods.

***Neural correlations with DDM Bias***

Testing for neural correlates of the DDM Bias parameters yielded no results that survived whole-brain correction (Table S4).

***Behavioral associations between tasks***

At an anonymous reviewer’s request, we tested whether there were behavioral associations between the tasks when correlating the task performance within the domain of appetitive / positive and aversive / negative stimuli.

The analysis in separate domains did not yield significant correlations either. For the correlation of positive emotion regulation success with the overall success level in refusing to eat palatable-unhealthy foods, Spearman’s rho was 0.19, Posterior Probability(Rho > 0) = 0.86, 95% HDI = [-0.13; 0.51]. For the correlation of the negative emotion regulation success with the overall success level in accepting to eat healthy-unpalatable foods, Spearman’s rho was -0.16, PP (Rho < 0) = 0.82, 95% HDI = [-0.50; 0.18].

**Supplementary Figures**

**Figure S1.** Reappraisal Success by trial type. This figure shows the mean reappraisal success for both types of reappraisal. Reappraisal success was the trial-wise difference of the rating given after regulation minus the rating given when just viewing the stimulus for negative stimuli, and vice versa for positive stimuli. The black solid line represents the group mean and the gray box indicates the standard error of the group mean. Each dot represents the mean reappraisal success by reappraisal type for one participant.

**
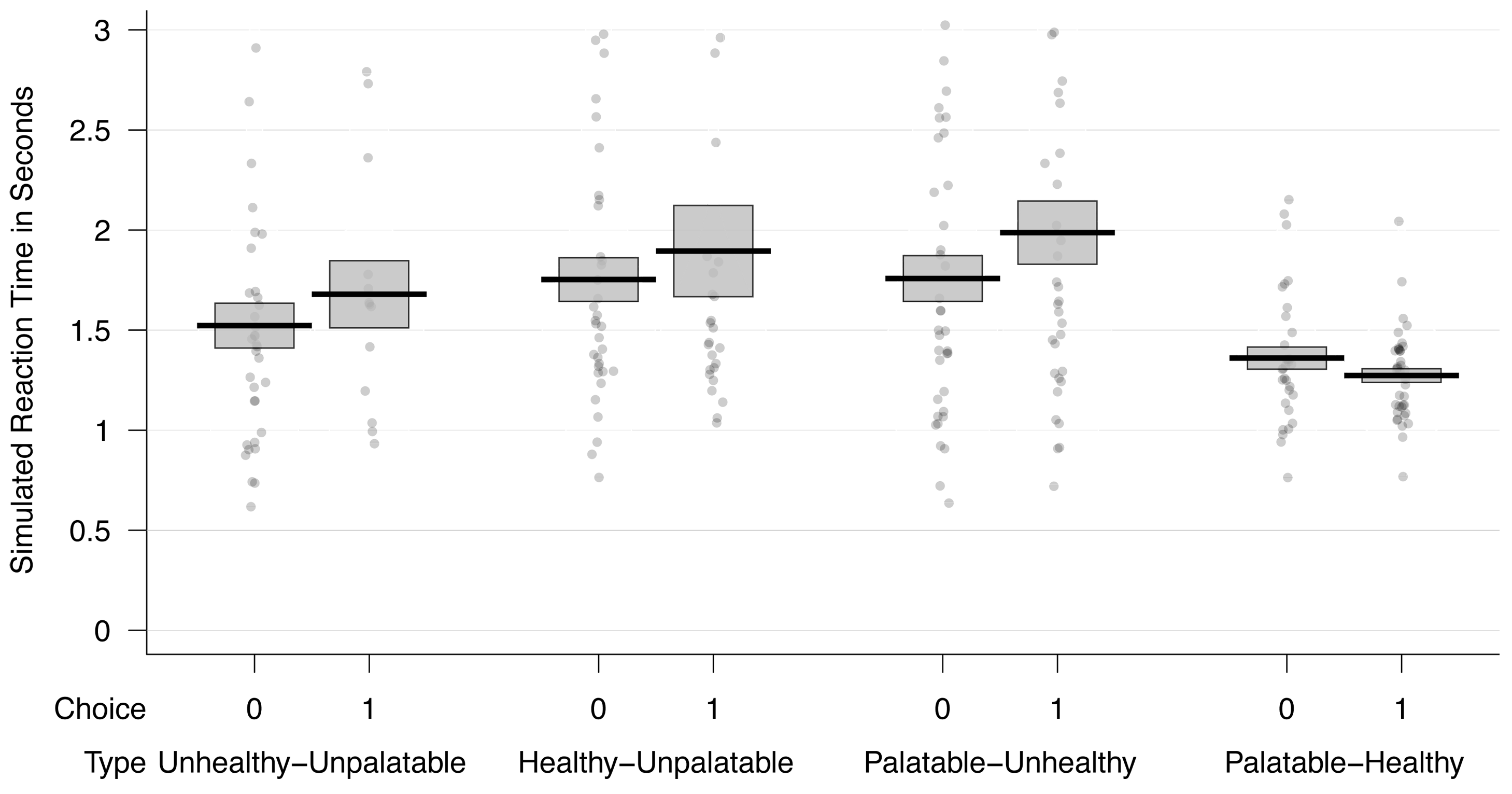
**

**Figure S2.** Simulated reaction times generated from the best-fitting DDM parameters (analogous to Figure 3 in the main text). This figure shows the mean reaction times (RTs) over all simulated participants for accepting (Choice = 1) and refusing (Choice = 0) to eat foods from each of the four categories. The black solid line represents the group mean and the gray box indicates the standard error of the group mean. Each dot represents the mean RT by choice category for one simulated participant. On average, the generating drift diffusion models, on which each participant’s simulations were based, had a starting point bias towards refusing to eat the foods. Qualitatively, this plot of the simulated reaction times for the different food categories captures the features of Figure 3 in the main text. This suggests that the results observed in the experiment are consistent with a starting-point-bias in favor of refusing the foods.

**
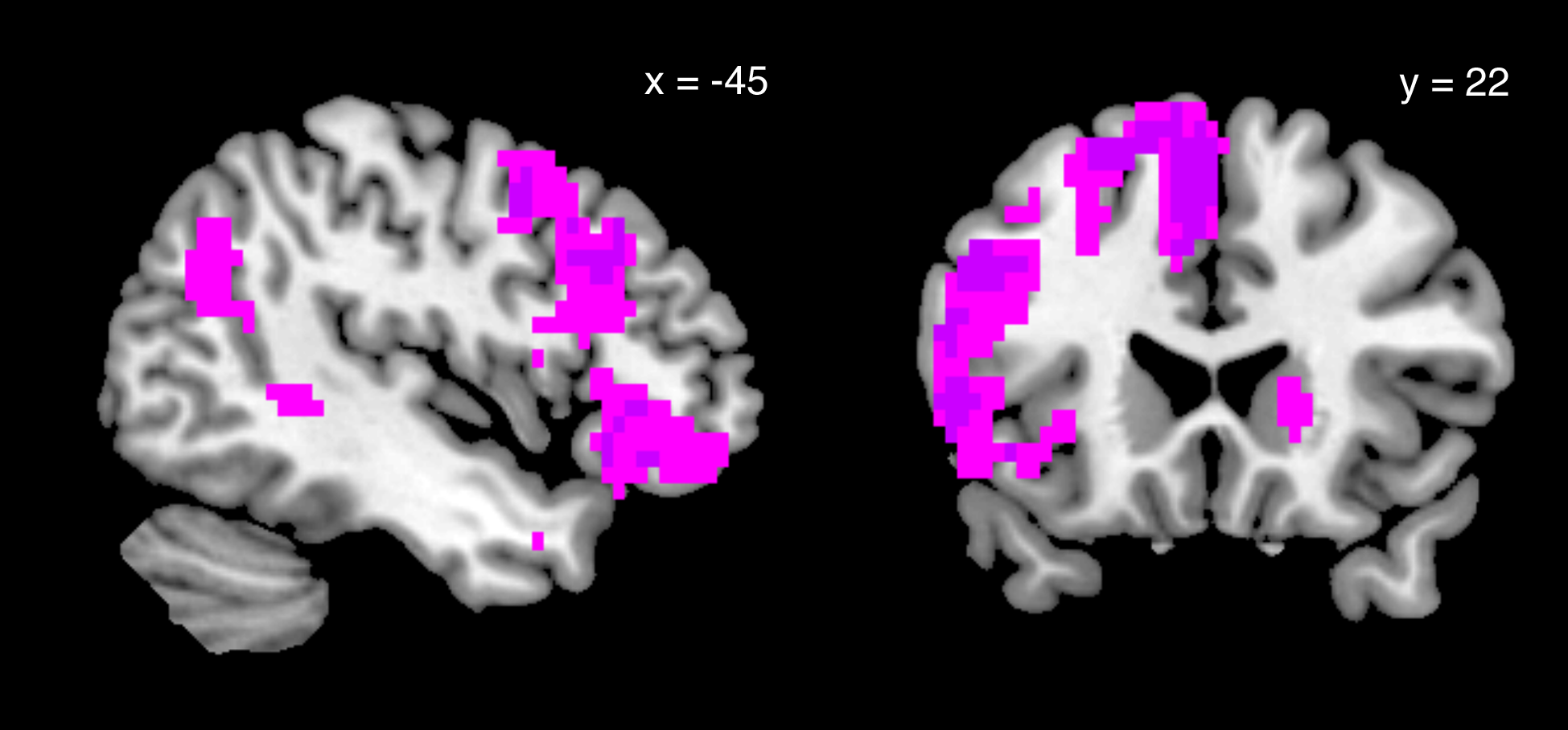
**

**Figure S3.** Whole-brain family-wise error corrected group-level results (p < 0.05) for the contrast Reappraise > View (violet, darker shading) and the contrast Reappraisal Success > View (pink, lighter shading) from GLM-ER. The two contrasts are very similar. This is not surprising because participants rarely failed to reappraise the image content.

**Supplementary Tables**

**Table S1.** Regression modeling positive versus negative reappraisal success.

| **Fixed  effects** | **Beta  estimate** | **Standard  Deviation** | **95 % Highest Density Interval** |
| --- | --- | --- | --- |
| **Intercept** | **1.64** | **0.14** | **[1.35; 1.92]** |
| Positive Reappraisal | 0.29 | 0.16 | [-0.02; 0.62] |
| **Bayesian R^2^** | **0.30** | **0.02** | **[0.27; 0.34]** |

This table reports the results from the Bayesian logistic regression model specified in Equation S1 explaining regulation success (i.e. the trial-wise difference of the rating given after regulation minus the rating given when just viewing the stimulus for negative stimuli, and vice versa for positive stimuli) by regulation *Type*. The intercept measures regulation success in the negative reappraisal condition that served as a baseline. The *Positive Reappraisal* term measures the difference between the positive and negative regulation condition. The regression included participant-specific intercepts and participant-specific random slopes for the factor *Type*. The coefficients (Beta estimates) listed are the means of the population level posterior distributions ± standard deviation (SD) and the 95% Highest Density Interval. The analysis comprised N = 36 participants.

**Table S2.** Time-varying DDM parameters.

| **Dataset** | **Parameter estimate** | | | | | |
| --- | --- | --- | --- | --- | --- | --- |
| *(1) ESC* | **ω_taste_** | **ω_health_** | **Thr** | **nDT** | **RST** | **Bias** |
| Current food  choices | 0.36 ± 0.40 | 0.41 ± 0.26 | 1.13 ± 0.27 | 0.72 ± 0.12 | -0.02 ± 0.43 | -0.37 ± 0.29 |
| *(2) IAC* | **ω_taste_** | **ω_health_** | **Thr** | **nDT** | **RST** | **Bias** |
| Natural  Choice | 1.37±  0.79 | 0.33±  1.33 | 1.27 ±  0.28 | 0.86 ±  0.12 | 0.42 ±  0.54 | 0 ±  0.37 |
| Health  Cued choices | 0.98 ±  1.12 | 1.11 ±  0.60 | 1.39 ±  0.36 | 0.85 ± 0.14 | -0.06 ±  0.55 | -0.22 ± 0.33 |

This table reports the group mean ( ± standard deviation) of the best-fitting parameters from a time-varying DDM model with separate attribute consideration onset times. The rows report the data from the current dietary self-control task (current food choices) and both the Health-cued and Natural (i.e. baseline) choices from a similar study by Hare et al. (2011) in which participants were given instructed attention cues (IAC) toward healthiness or palatability. Abbreviations:
**ESC**: the food choice data from the current study (Emotion regulation and dietary self-control). All food choices in this study were made under the instruction to consider healthiness and try to make healthy choices.
**IAC**: the food choice data from the instructed attention cue study by Hare et al. (2011). In the Health Cue” condition, participants were asked to focus on the health attributes of the presented food when making their choices, whereas in the “Natural Choice” condition, they were asked to choose as they naturally would do.

$\boldsymbol{\omega}_{\boldsymbol{taste}}$= weighting factor determining how much the difference in taste attributes contributes to the evidence accumulation rate.

$\boldsymbol{\omega}_{\boldsymbol{health}}$=weighting factor determining how much the difference in health attributes contributes to the evidence accumulation rate.

**Thr** = evidence threshold for responding.

**nDT**=non-decision time and corresponds to the starting time for taste in our model.

**RST**: relative start time for health (timing relative to start of taste processing, positive values denote that health enters the process later than taste).

**Bias**: starting point bias for the evidence accumulation process (zero = no bias).

**Table S3.** Results of the Reappraisal > View contrast from GLM-ER.

| *Region* | *Side* | *MNI Coordinates* | *TFCE t-stat* |
| --- | --- | --- | --- |
| Superior Frontal Gyrus | L | -5 12 61 | 6.07 |
| Inferior Frontal Gyrus, pars triangularis | L | -53 27 18 | 5.02 |
| Middle Frontal Gyrus | L | -35 4 61 | 5.05 |
| Middle Frontal Gyrus | L | -48 24 32 | 5.81 |
| Middle Temporal Gyrus, posterior division | L | -53 -44 0 | 6.8 |
| Superior Frontal Gyrus | L | -5 47 43 | 6.24 |
| Middle Frontal Gyrus | L | -45 17 40 | 4.29 |

This table reports the results of the contrast Reappraisal > View, collapsed across both positive and negative valence in order to test for domain-general regulation mechanisms. It is analogous to Table 5, except that it includes all reappraisal block trials regardless of success or failure. All reported regions were significant at p < .05 after whole brain family-wise error correction. Threshold free cluster enhancement (TFCE) test statistics and their null distribution (5000 permutations) were calculated with the Randomise package in FSL. Anatomical labels were derived from the Harvard-Oxford cortical and subcortical atlases. The analysis comprised N = 35 participants.

**Table S4.** BOLD signals were not significantly correlated with starting point biases.

| ***(1) Self-Control Success > Self-Control Failure*** | ***DDM parameter*** | ***Direction of correlation*** | **Minimum p-value** |
| --- | --- | --- | --- |
|  | Bias | positive | 0.25 |
|  | Bias | negative | 0.83 |
| ***(2) All Food Choices*** |  |  |  |
|  | Bias | positive | 0.50 |
|  | Bias | negative | 0.85 |

We conducted a set of exploratory analyses to see if starting-point bias parameters from the DDM correlated with BOLD activity in any voxels at the time of choice. We used two contrasts, 1) Self-Control Success > Self-Control Failure contrast from GLM-SCS , and 2) the All Choice contrast from GLM-FC. There were no significant correlations after correcting for multiple comparisons at the whole brain level. Here, we report the brain-wide minimum p-value for each contrast and correlation direction. The correlations with *All Food Choices* comprise N = 37 participants. The correlations with *Self-Control Success > Self-Control Failure* comprise N = 35 participants because this contrast could not be evaluated for two participants due to too few failures.

**References**

Hare TA, Malmaud J, Rangel A (2011) Focusing attention on the health aspects of foods changes value signals in vmPFC and improves dietary choice. The Journal of neuroscience : the official journal of the Society for Neuroscience 31:11077-11087.

Kruschke JK (2013) Bayesian estimation supersedes the t test. J Exp Psychol Gen 142:573-603.

Maier SU, Beharelle AR, Polania R, Ruff CC, Hare TA (in press) Dissociable mechanisms govern when and how strongly reward attributes affect decisions. Nature Human Behaviour.
